# Prevalence of immunoglobulin G (IgG) antibody to *Neospora caninum* in dairy cattle of Hamedan province, west of Iran

**Published:** 2014

**Authors:** Jamal Gharekhani, Hamidreza Haddadzadeh, Alireza Bahonar

**Affiliations:** 1 *Department of Parasitology, Central Veterinary Laboratory, Iranian Veterinary Organization, Hamedan, Iran; *; 2 *Department of Parasitology, Faculty of Veterinary Medicine, University of Tehran, Tehran, Iran; *; 3 *Department of Food Hygiene & Quality Control, Faculty of Veterinary Medicine, University of Tehran, Tehran, Iran.*

**Keywords:** Cattle, Hamedan, Iran, *Neospora caninum*, Prevalence

## Abstract

Bovine neosporosis caused by the apicomplexan protozoan parasite *N. caninum*, was initially recognized in 1989 and is now reported as a leading infectious cause of reproductive failure in dairy cattle in world wide. The aim of this study was to determine the seroprevalence of *N. caninum* infection in industrial dairy cattle of Hamedan province (west of Iran) by ELISA method. Blood samples were collected from 492 cattle in 41 farms. Antibodies to* N. caninum* were found in 63(12.80%) sera. A Significant difference was observed between seropositive cattle and dog presence in farm, dog contact with herd, abortion history and herd population. No significant differences were found between seropositive cattle and age as well as breed. This study is the first report of *N. caninum* infection in dairy cattle farms in Hamedan province. As per our knowledge,* Neospora* is an important factor in abortion of cattle in this region. Therefore, comprehensive studies for control strategies and improving management of dairy farms is necessary.

## Introduction

*Neospora caninum* is a protozoa that causes abortion and economic losses in cattle worldwide.^1^ In cattle, transplacental transmission is the main mechanism by which the parasite persists in a herd.^2^ After recognizing the dog as the definitive host of the parasite, epidemiological work established the association between the presence of dogs and the disease in cattle.^3^^,^^4^ Additionally, the association of canids with cattle on their premises, has been postulated as a risk factor for the disease.^5^ Similarly, it has been established that intensive herd management was associated with increased seroprevalence to *N. caninum*.^6^ The presence of wild canids have also been related with high prevalence of sero-active cattle.^7^ Neosporosis of cattle has been associated with abortion, neonatal mortality and decrease in the volume of milk production that cause yearly economic loss.^8^

The seroprevalence of *N. caninum* infection in cattle varies largely, depending on the country and region.^8^ Several assays are available for detecting antibodies to *N. caninum* in cattle.^9^^,^^10^ Some serological studies in dairy herds have done in some part of Iran. However, there is not published information of *N. caninum* infection in the cattle of this province. 

This study was performed to determine the prevalence of antibodies to *N. caninum* in industrial dairy cattle in Hamedan province, using enzyme-linked immunosorbent assay (ELISA).

## Materials and Methods

A cross-sectional study was performed in the first half of year 2010. Blood samples were taken from 492 dairy cattle in the 41 industrial farm of Hamedan province, Iran. The animals were randomly selected. The owners were questioned about age, breeding, dog presence and its contact with the herds, abortion history, and herd population.

All samples were immediately transported to the diagnostic laboratory of Hamedan Veterinary Office, Hamedan, Iran. Serum was removed after centrifugation at 1000 *g* for 15 min. All sera were stored at -70 ˚C until laboratory testing.^8^

The samples were analyzed for antibodies against *N. caninum* using ELISA kit. Anti-*Neospora* antibodies were detected using a commercially available *N. caninum* ELISA kit (Herdcheck, Maine, USA). The kit was used according to the manufacturer’s instructions. The presence or absence of antibody was determined by calculating of sample to positive ratio (S/P ratio according to the formula mentioned inside the manual). A S/P ratio more than 0.5 and less than 0.5 was considered positive and negative, respectively.

## Results

Immunoglobulin G (IgG) antibodies to *N. caninum* were found in 63 of 492 (12.80%) sera (CI = 0.12 ± 0.03). With regard to seropositivity, significant differences were found regarding herd population (X^2 ^= 13.15, df = 1.00 and *p *< 0.001), abortion history (X^2 ^= 48.06, df = 1.00 and *p* < 0.001), dog presence in farm (X^2 ^= 9.45, df = 1.00 and *p* = 0.002), dog contact with herd (X^2 ^= 5.73, df = 1.00 and *p* = 0.01); and stray canids presence in farm (X^2^ = 37.17, df = 1.00 and *p* < 0.001). There were no significant differences between seroprevalence and age (X^2^ = 3.96, df = 3.0 and *p* = 0.262), as well as breeding (X^2^ = 0.88, df=1.00 and *p* = 0.346), (Table 1).

**Table 1:** Comparison of *N. caninum* seroprevalence in different variables

	**Age groups**				**Breed**		**Herd capacity**		**Dog presence in** **farm**	**Dog** **contact with herd**	**Stray canids presence in farm**	**Abortion history**	**Total**
	**>** **2**	**2** **-** **3**	**3** **-** **4**	**<** **4**	**Hybrid**	**Holstein**	**≤** ** 100**	**< 100**					
**Number of sample (%)**	55 (11.20)	113 (23.0)	147 (29.90)	177 (36.00)	38 (7.70)	454 (92.30)	216 (43.90)	276 (56.10)	360 (73.20)	192 (53.30)	144 (29.30)	35 (7.11)	492 (100)
**Number of positive (%)**	11 (20.00)	15 (13.30)	14 (9.50)	23 (13.00)	3 (7.90)	60 (13.20)	41 (19.00)	22 (8.00)	36 (10.00)	26 (13.50)	39 (27.00)	20 (57.10)	63 (12.80)

## Discussion

This study was the first report of *N. caninum* infection in industrial dairy cattle in Hamedan proviance. There were only a few reports on* N. caninum* seroprevalence in dairy cattle of Iran.^10^^-^^13^ The seroprevalence rate were reported 32.00% in Babol (north of Iran), 46.00% in Mashhad (northeast of Iran) and 12.60% in Kerman (southeast of Iran) using ELISA.^10^^-^^12^ The similar rate of infection was reported in Brazil, Greece, Peru, Australia, Canada, Ireland, Korea and Spain. ^3^^,^^9^^,^^14^

In the present study, the herd seroprevalence was similar to that study in Thailand and different to other countries.^3^^,^^13^ Difference of management in farms, study design and sample size are main cause of varied results.

In current study, there was no significant difference in seroprevalence between the different age groups which was similar to result of Nourollahi *et al*. in Kerman and other researchers.^9^^,^^10^^,^^15^^-^^18^ Razmi *et al*. reported statistically significant in different age groups.^12^ Sadrebazzaz *et al*. and Wouda *et al*. reported equal levels of seroprevalence in all age groups for most herds.^13^^,^^19^ Jensen *et al*. suggested seroprevalence increased with age and depended on sample size.^20^ Lower seroprevalence in cattle of > 2 age is due to a decrease of antibody in congenital infections. It seems relationship between age and seroprevalence rate is speculative.

In a French study, similar to our study, there was not association between seropositivity and breed.^21^ The prevalence of *N. caninum* in dairy cattle was reported higher than beef cattle in Spain.^22^^,^^23^ This might be related to different production systems for dairy and beef cattle rather than to breed differences. Comprehensive research on the impact and role of different breed in the prevalence of infection is essential.

In this study, there was a 2.70 fold increase of seroprevalence in farms with more than 100 individuals [*p* = 0.0005, OR = 2.70(1.55-4.70)]. Kyaw *et al*., reported that cattle in larger farms (≥ 21) had a higher infection than small herds (< 21) in Thailand, (*p* = 0.03); opposite to Davison *et al*. study.^17^^, ^^24^

In a study in Italy, the risk of seropositive case increased with the herd size due to increasing number of dogs.^25^ Our results provide strong support for the hypothesis that increase of herd capacity is a risk factor of *Neospora* infection.

In present study, 57.10% of cattle with abortion history were seropositive (*p* = 0.0005). Razmi *et al*. reported, that the abortion prevalence in seropositive cattle was higher than seronegative (*p* < 0.05, OR = 1.78) in Mashhad.^12^ This is similar to our and other results.^2^^,^^3^^,^^9^^,^^18^ Evaluation of seropositivity in previous studies showed that the risk of abortion were 4.00, 5.30 and 8.00 fold higher than seronegative cattle.^26^^-^29 Our result taken together with previous investigations supports the notion that the seropositivity rate is correlated with abortion.

In current study, *N. caninum* infection was reported 10.00% (36/360) in the farms with dog presence [*p *= 0.002, OR = 0.43(0.25-0.74)]. However, 13.50% (26/122) of cattle in contact with dog were seropositive. A 2.47 fold increase in the rate of infection was observed in cattle with dog contact [*p* = 0.02, OR = 2.47(1.16-5.30)]. Around 27.00% (39/144) of cattle in contact with stray canids (fox and jackal) were seropositive (*p* = 0.0005).

Studies in Spain and France have also found positive associations between the seropositivity of cattle and the presence or the number of farm dogs.^21^ Barling *et al*. observed the presence of dog in farms was a putative protective factor.^30^ Our result is the opposite of Kyaw *et al*. finding.^17^

The presence of dogs in farm has been assumed to provide the greatest chance of horizontal transmission through the ingestion of oocysts shed by infected dogs. In addition, dogs kept in the neighborhood farms may pose an infection risk.

The results of this research can provide baseline information for the future studies. There are both horizontal and vertical transmissions of *N. caninum* in Hamedan province. Therefore, evaluation of *Neospora* infection in other intermediate hosts and also definitive hosts are necessary for control strategies.

In conclusion, *N. caninum* is an important factor of economic loss in industrial dairy cattle in Hamedan province. Therefore, it warrants a complete overhaul of management in dairy farms.

## References

[1] Dadin M, Carlos C, Anselmo O (2002). Seroepidemiology of beef and dairy herds and fetal study of N caninum in Argentina. Vet Parasitol.

[2] Anderson ML, Andrianarivo AG, Conrad PA (2000). Neosporosis in cattle. Anim Reprod Sci.

[3] Dubey JP, Schares G (2011). Neosporosis in animals - The last five years. Vet Parasitol.

[4] Wouda W, Dijkstra TH, Kramer AM (1999). Seroepidemiological evidence for a relationship between N caninum infections in dogs and cattle. Int J Parasitol.

[5] Mc Allister MM, Bjorkman C, Anderson R (2000). Evidence of point-source exposure to N caninum and protective immunity in a herd of beef cows. J Am Vet Med.

[6] Sanderson MW, Gay JM, Baszler TV (2000). N caninum sero-prevalence and associated risk factors in beef cattle in northwestern United States. Vet Parasitol.

[7] Barling KS, Sherman M, Peterson MY (2000). Spatial association among density of cattle, abundance of wild canids and seroprevalence to N caninum in a population of beef calves. J Am Vet Med.

[8] Salehi N, Haddadzadeh HR, Shayan P (2010). Serological study of N caninum in pregnant cattle in Tehran, Iran. Int J Vet Res.

[9] Dubey JP, Schares G, Ortegamora LM (2007). Epidemiology and control of Neosporosis and N caninum. Climic Rev.

[10] Nourollahifard SR, Khalili M, Aminzadeh A (2008). Prevalence of antibodies to N caninum in cattle in Kerman province, South East Iran. Vet Archiv.

[11] Yousefi MR, Arabkhazaeli F, MollaHassan T (2009). Sero-prevalence of N caninum infection in rural and industrial cattle in northern Iran. Iranian J Parasitol.

[12] Razmi GR, Mohammadi GR, Garrosi T (2006). Sero-epidemiology of N caninum infection in dairy cattle herds in Mashhad area, Iran. Vet Parasito.

[13] Sadrebazzaz A, Haddadzadeh H, Esmailnia K (2004). Serological prevalence of N caninum in healthy and aborted dairy cattle in Mashhad, Iran. Vet Parasitol.

[14] Meerschman F, Focant C, Boreux R (2000). Cattle neosporosis in Belgium: A case-control study in dairy and beef cattle. Int J Parasitol.

[15] Atkinson RA, Cook RW, Reddacliff LA (2000). Sero-prevalence of N caninum infection following an abortion outbreak in a dairy cattle herd. Aust Vet J.

[16] Chanlun A, Näslund K, Aiumlamai S (2002). Use of bulk milk for detection of N caninum infection in dairy herds in Thailand. Vet Parasitol.

[17] Kyaw T, Virakul P, Muangyai M (2004). N caninum seroprevalence in dairy cattle in Central Thailand. Vet Parasitol.

[18] Paré J, Fecteau G, Fortin M (1998). Seroepidemiologic study of N caninum in dairy herds. J Am Vet Med.

[19] Wouda W, Moen AR, Schukken YH (1998). Abortion risk in progeny of cows after a N caninum epidemic. Theriogenology.

[20] Jensen AM, Bjorkman C, Kjeldsen AM (1999). Associations of N caninum seropositivity with gestation number and pregnancy outcome in Danish dairy herds. Prev Vet Med.

[21] Ould Amrouche A, Klein F, Osdoit C (1999). Estimation of N caninum seroprevalence in dairy cattle from Normandy, France. Vet Res.

[22] Quintanilla-Gozalo A, Pereira-Bueno J, Tabare E (1999). Seroprevalence of N caninum infection in dairy and beef cattle in Spain. Int J Parasitol.

[23] Hemphil A, Gottestin BA (2000). Europian perspective on N caninum. Int J Parasitol.

[24] Davison HC, Otter A, Trees AJ (1999). Estimation of vertical and horizontal transmission parameters of N caninum infections in dairy cattle. Int J Parasitol.

[25] Otranto D, Lazari A, Testini G (2003). Seroprevalence and associated risk factors of neosporosis in beef and dairy cattle in Italy. Vet Parasitol.

[26] López-Gatius F, Santolaria P, Almeria S (2005). N caninum infection does not affect the fertility of dairy cows in herds with high incidence of Neospora associated abortions. Vet Public Health.

[27] Schares G, Bärwald A, Staubach C (2004). Potential risk factors for bovine N caninum infection in Germany are not under the control of the farmers. Parasitol.

[28] Vaclavek P, Koudela B, Modry D (2003). Seroprevalence of N caninum in aborting dairy cattle in the Czech Republic. Vet Parasitol.

[29] Jenkins M, Baszler T, Bjorkman C (2002). Diagnosis and seroepidemiology of N caninum associated bovine abortion. Int J Parasitol.

[30] Barling KS, Mc-Neill JW, Paschal JC (2001). Ranch-management factors associated with antibody sero-positivity for N caninum in consignments of beef calves in Texas. USA Prev Vet Med.

